# Computed Energetics of Nucleotides in Spatial Ribozyme Structures: An Accurate Identification of Functional Regions from Structure

**DOI:** 10.1100/tsw.2004.19

**Published:** 2004-03-26

**Authors:** Ivan Y. Torshin

**Affiliations:** Federal Science Center (VEI), Moscow, Russia

**Keywords:** ribozymes, catalytic site, molecular mechanics, structure-function relationship

## Abstract

Ribozymes are functionally diverse RNA molecules with intrinsic catalytic activity. Multiple structural and biochemical studies are required to establish which nucleotide bases are involved in the catalysis. The relative energetic properties of the nucleotide bases have been analyzed in a set of the known ribozyme structures. It was found that many of the known catalytic nucleotides can be identified using only the structure without any additional biochemical data. The results of the calculations compare well with the available biochemical data on RNA stability. Extensive *in silico* mutagenesis suggests that most of the nucleotides in ribozymes stabilize the RNA. The calculations show that relative contribution of the catalytic bases to RNA stability observably differs from contributions of the noncatalytic bases. Distinction between the concepts of “relative stability” and “mutational stability” is suggested. As results of prediction for several models of ribozymes appear to be in agreement with the published data on the potential active site regions, the method can potentially be used for prediction of functional nucleotides from nucleic sequence.

### INTRODUCTION

Ribozymes are functionally diverse RNA molecules with intrinsic catalytic activity[1]. Although known natural ribozymes catalyze phosphodiester-transfer chemistry, RNA molecules that catalyze formation of nucleotide from sugar and base[2], synthesize amide bonds[3,4], and even synthesize acyl-coenzyme A[5] were also identified. The naturally occurring ribozymes have been studied extensively and a number of structures of the ribozymes have been solved[1,6,7,8,9,10,11,12,13]. The chemical mechanism of a ribozyme (and, in particular, the bases involved in the catalysis) is not apparent even if a solved structure is available. To establish functionally important bases, many additional mutational and other biochemical studies are required. Biochemical data on modification of functional groups that result in decreased activity can be correlated with particular type of tertiary interactions observed in a structure (such as non-

Watson-Crick base pairing observed in "hammerhead" ribozyme[9]) or surface accessibility plots, but only in some cases. Thus, a computational method to predict potential functional bases from the solved structure (or using a structural model of a ribozyme) could be a useful tool for the actual researchers in the area of macromolecular structure and function.

Such a method was elaborated and has been successfully applied to a few ribozymes with known structures (hairpin, hammerhead and group I intron) [14]. The results of calculations [14] compare well with the mutational data for these ribozymes. The idea of this apparently successful method has been borrowed from the extensive studies made on the protein enzymes and general physical chemistry. Transition state theory is a general physico-chemical concept that provides a number of valuable explanations for homogenous, heterogeneous and enzymatic catalysis. The theory is general enough to be applicable to the RNA enzymes too. In particular, application of the theory to enzymes (that is, proteins with catalytic sites) suggests that the energetic barrier is likely to be reduced by the ground state destabilization. Electrostatic effects would provide an important contribution to enzyme catalysis[15]. These general considerations are confirmed by a number of theoretical and biochemical studies of individual protein enzymes[16,17,18,19,20,21,22] and can be summarized as follows: Relative physicochemical properties of the functional residues of the enzymes are very distinct from the other residues in a molecule. Now, this is the central idea of the present method: If some relative physicochemical property can be calculated from a structure of a ribozyme, then the bases involved in catalytic site can be identified. Functional residues destabilize proteins/enzymes[16,17,18,19] and the energetic contribution of individual residues can be reliably calculated ([14,20,21] and unpublished data). It is also known that catalytic residues in enzymes also have unusual regions on theoretical microscopic titration curves[22]. Thus, once again, catalytic residues are distinct from all of the remaining residues in an enzyme[22]. For proteins with known spatial structure, the functional residues can be predicted using these theoretical methods[21,22].

In the present study, the relative energetic properties of the individual nucleotides in the solved ribozyme structures have been analyzed. Although the stronger electrostatic interactions are important for enzyme function and protein stability[15,20,21], the weaker Van der Waals interactions should not be neglected in calculating the contribution of individual residues/bases to stability of the whole molecule. The full molecular mechanics potential that included bond, angle, torsional, hybrid, and nonbonding (electrostatic and Van der Waals) energy terms has been used to analyze the energetic properties of the bases in the known spatial structures of ribozymes taken from PDB[23]. To take into account the Van der Waals interactions, the "continuum electrostatics calculations" (such as described in [21]) are not applicable and the most plausible technique will be molecular mechanics calculations. Using molecular dynamics within the range of the classical molecular mechanics techniques also allows sampling of the conformational space necessary to take into account an entropic component of the free energy.

Molecular mechanics calculations were performed using ECMMS (described in Appendix I) — a library of computational routines specifically developed by the author for the purposes of the study. The ECMMS routines use UFF force field parameters and potentials[24] for bonding, angular and torsional terms, and AMBER scheme[25] for nonbonding terms. The reasons for combining the UFF and AMBER force fields are also mentioned in Appendix I. These calculations were performed using a "multiple trajectories" technique[26,27] that is more likely to produce models closer to experimental structures than simply an energy minimization or a singular long trajectory of molecular dynamics. Apart from NMR and crystal structures of ribozymes[1,6,7,8,9,10,11,12,13,28,29,30,31,32], the method was also applied to RNAs of other types[33,34,35] as well as to several structural models of ribozymes[36,37,38].

An important point to be considered is the measures of stabilization provided by some or other base in a RNA molecule. Unlike proteins, the experimental data on the effects of mutations on the RNA stability are rather scarce. No extensive mutagenesis has been done even of a single ribozyme while a number of "alanine-scanning" studies were done for proteins (for example, for lysozyme[39,40]). Moreover, in proteins substitutions like Arg→Ala or Asp→Ala are substitutions of a "wild-type residue" (referred to further as a "native residue") for a smaller residue with distinctly different physicochemical properties. Such substitutions are of "side-chain deletion" type and thus can ascertain unequivocally contribution of a native Arg or Asp etc. to the stability of the native structure (some of such valuable biochemical data for proteins are summarized in [21]). In RNA, however, there are mainly four nucleotides of similar size and similar physicochemical properties. Therefore, results of RNA mutagenesis done for the study of the contribution of individual bases to RNA stability would be more ambiguous (this point is discussed in detail further in this article). In addition, a mutation of the individual bases is likely to disrupt the native packing (especially packing in the CG, AU base pairs) and, therefore, it can be said *a priori* that a majority of RNA mutants would, generally, destabilize RNA. Indeed, the mutational studies of selected bases in ribozymes[41,42,43,44,45] show that mutating a native base in a ribozyme to another base mostly destabilizes the RNAs. These three points (scarcity of the experimental data, ambiguity of the mutational stability of RNA, and *a priori* destabilization of RNA mutants) suggest that it might seem difficult to correlate data on mutational stability of RNA with the “relative energetic contribution” described here that each native base provides to the stability of the native ribozyme. In this work, we show that results of *in vitro* biochemical mutagenesis of a ribozyme can be quantitatively correlated with *in silico* computational mutagenesis of the structural model of the ribozyme: td (thymidylate synthase) intron of bacteriophage T4. We also present the results of an exhaustive single-base mutagenesis *in silico* for a number of small ribozymes to make a clear distinction between “mutational” and “relative” measures of contribution of a nucleic base to the RNA stability.

### MATERIALS AND METHODS

#### RNA Structures

The structures of 8 ribozymes of various structure and sizes[6,7,8,9,10,11,12,13], from 24 residue hairpin[6] to ~500 residue 23S ribosomal catalytic fragment[13], were taken from PDB[23]. The data on the known functional residues are summarized in Table 1 and graphically presented in Figure 1 to orient the reader.

**TABLE 1**
**The Known Functional Nucleotides in the Ribozymes**

| PDB | Data | RNA Type | Nr. | Active Site | Substrate-Binding | Mg-Binding | Ref |
|---|---|---|---|---|---|---|---|
| 1e4pA | NMR | Hairpin | 24 | A7,G8,C18,G19, **clv:**G5,A6 | — | — | [6] |
| 1ldz_ | NMR | Leadzyme | 30 | C6,G7,G9,C10,G23,G24,A25 | — | — | [8] |
| 1hmhA | X-ray | Hammerhead | 34 | G10.1,G5,G8,A14 | — | A9,A13,A14 | [9] |
| 300dA | X-ray | Hammerhead | 41 | G5,A6,U16.1,C3 | — | G10.1,A9,U7,C1.2 | [10] |
| 1m5kB | X-ray | Hairpin | 92 | G8,A9,A10,A24,A38 [32]<br>G21,A22,A23,C25 [31] | — | — | [32] |
| 1cx0B | X-ray | HDVribozyme | 94 | U120,C122,G125,G138,G139, G140 | — | — | [11]*1 |
| 1grzA | X-ray | Group I intron | 246 | A207,C208,A261,A306 | — | — | [12] |
| 1fg0A | X-ray | 23S Ribosomal subunit | 496 | G2102,G2482,A2485,A2486, G2588,U2619,U2620 | C2542,U2589, U2590,C2608, G2618 | — | [13] |

*1 A178, C175 said to be catalytic[11] are not in the structure (PDB: 1cx0).

*Note:* The entries are ordered according to the ribozyme size. **nr**, number of residues in the PDB file; **clv:** cleavage site. Residue numeration of the original papers was preserved.

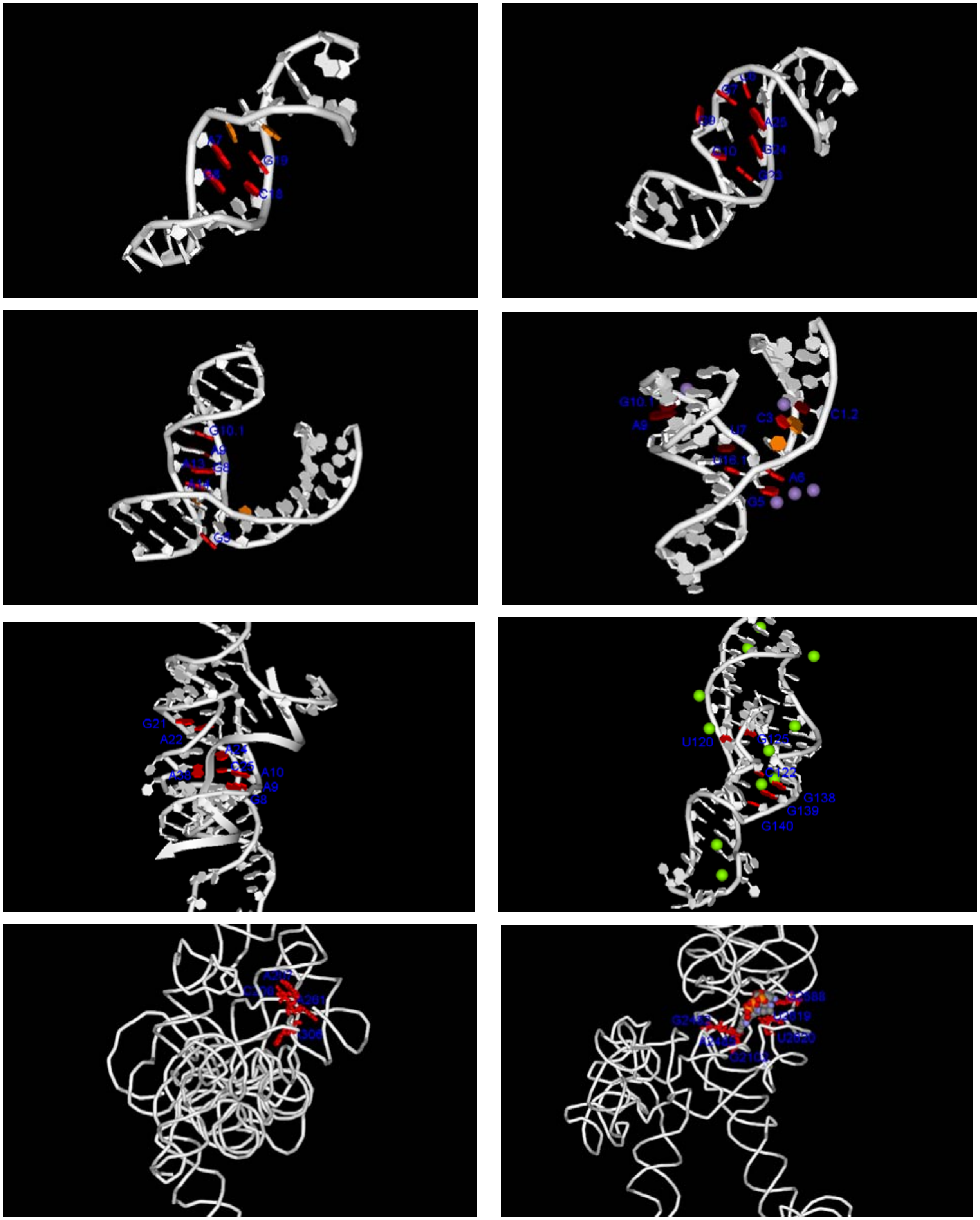

**FIGURE 1**. Active site regions in the ribozymes analyzed in this study. Known catalytic nucleotides (red), cleavage sites (orange), and metal ions (spheres) are shown. Nucleotides are numbered according to the original papers. The slides were made with WebLab Viewer (MSI corp, CA, USA; http://www.msi.com). (a) PDB 1e4p, "hairpin"; (b) PDB 1ldz/2ldz, leadzyme; (c) PDB 1hmh, "hammerhead", substrate strand is shown; (d) PDB 300d, "hammerhead", metal-binding nucleotides are in dark red; (e) PDB 1m5k, "hairpin", substrate strand is shown as a ribbon; (f) PDB1cx0, HDV ribozyme, role of the metal ions in the catalysis is not known; (g) PDB 1grz, group I intron, putative active site region is shown; (h) PDB 1fg0, catalytic core of 23S ribosomal subunit, a substrate analog is shown.

### Molecular Modeling

Detailed description of the ECMMS procedure used for energy calculations is given in the Appendix I along with the printout of the procedure. The calculations used “swarm” or “multiple trajectory” technique[26,27]. Calculations were made without hetero-atoms using either explicit or implicit modelling of solvent. Minimization (100 steps, conjugate gradients) was intermingled by short (1 ps) trajectories of molecular dynamics (MD) to achieve a stable energy minimum[25]. Using different sets of initial velocities, 10 parallel trajectories of this minimization procedure were made, then the 10 resulting structures were averaged and the average structure was minimized. Results are summarized in Table 2 and illustrated by Figs. 2–4.

**TABLE 2**
**The Least Stabilizing Nucleotides in Ribozymes**

| PDB | RNA | Known Functional Nucleotides | Adjacent to Active Site | Other, “False Positives” |
|---|---|---|---|---|
| 1e4pA | Hairpin | G8,C18,G19,G5,A6 | A9 | A20 |
| 1ldz_ | Leadzyme | G7,G23,G24,A25 | A8,G26 | G3 |
| 1hmhA | Hammerhead | G5,G8,A9,G10.1 | A6,U7 | G11.4,G11.3 |
| 300dA | Hammerhead | G5,A6,U16.1,U7,A9 | G16.2,G8 | G12-A14 |
| 1m5kB | Hairpin | A23,A24,C25,A38 | A40,U41,U42 | C13,A14,A15,U39 |
| 1cx0B | HDVribozyme | U120,C122,G138 | G101,C121,C163,A165 | G129,G131,G164,A166 |
| 1grzA | Group I intron | A207,A261,A306 | G112,U305 | A183,U185,G212,G215,G254,A256, G257,G272,G329 |
| 1fg0A | 23S Ribosomal subunit | G2102,G2482,A2485,A2486, U2620; mg: U2542, C2608 | C2105,U2107,G2284,U2484, C2487,A2488,A2532,U2535; U2527,U2529, U2541, G2543,A2637 | G2073,G2279,G2280,G2281,G2285, G2293,G2462,A2463,G2471,U2545, G2605,G2606,G2609 |

*Note:* Residue numeration of the original papers was preserved. Atoms of the “adjacent to active site” nucleotides were less than 4 Å from the known residues of an active site; these residues were typically in the active site cleft. Due to much larger number of nucleotides in 1fg0A (catalytic fragment of 23S ribosome subunit), the data presented here are for 0.95 $E_{max}$ cut off instead of the “standard” 0.9 $E_{max}$.

### Identification of the Least Stabilizing Residues

The contributions of individual residues to stability were calculated using the full molecular mechanics potential (UFF + AMBER[24,25], Appendix I) that treats both the electrostatic and Van der Waals interactions. The energy values for each individual base were obtained using the “E_residue" procedure of ECMMS, then the energy histograms (such as in Fig. 2) were drawn. The free energy values were calculated as dG = dH-TdS(configurational)-TdS(solvation). The configurational component of the entropy was taken into account by using the parallel trajectories of molecular dynamics simulations with the subsequent averaging of the structures (as described above). The solvent component was taken into account either implicitly or explicitly. Implicit treatment has included using electrostatic screening potential[46,47,48] coupled with reduced net charges on phosphate groups[47] to imitate the effects of counter-ion screening and dielectric constant of 4. Explicit solvent model included solvent molecules and counter-ions. As the terminal residues (the first residue and the last residue) in proteins[21] and in RNA/DNA (unpublished data) systematically appear to have higher energies than the rest of the residues, the energy values for these two residues in each RNA were not calculated. For implicit solvent model, an empirical energy cut off of 0.9 $E_{max}$ (0.9 of the maximal per-residue energy for the molecule) was used for selecting the residues having the highest calculated energies per-residue. For explicit solvent the cut off was 2 $E_{max.}$

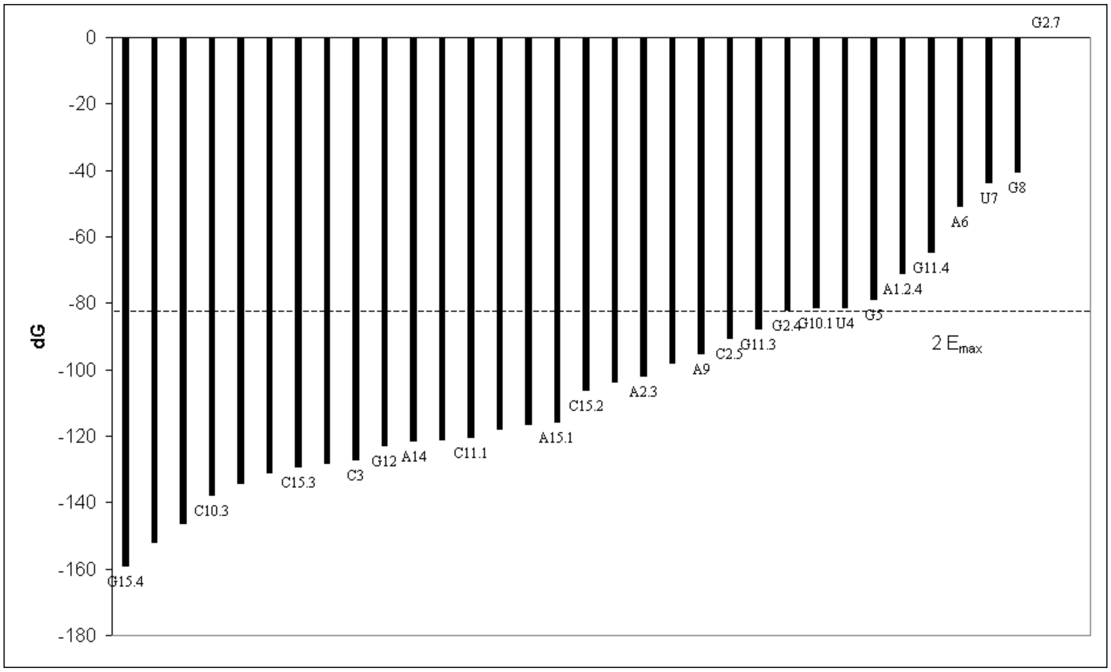

**FIGURE 2a**

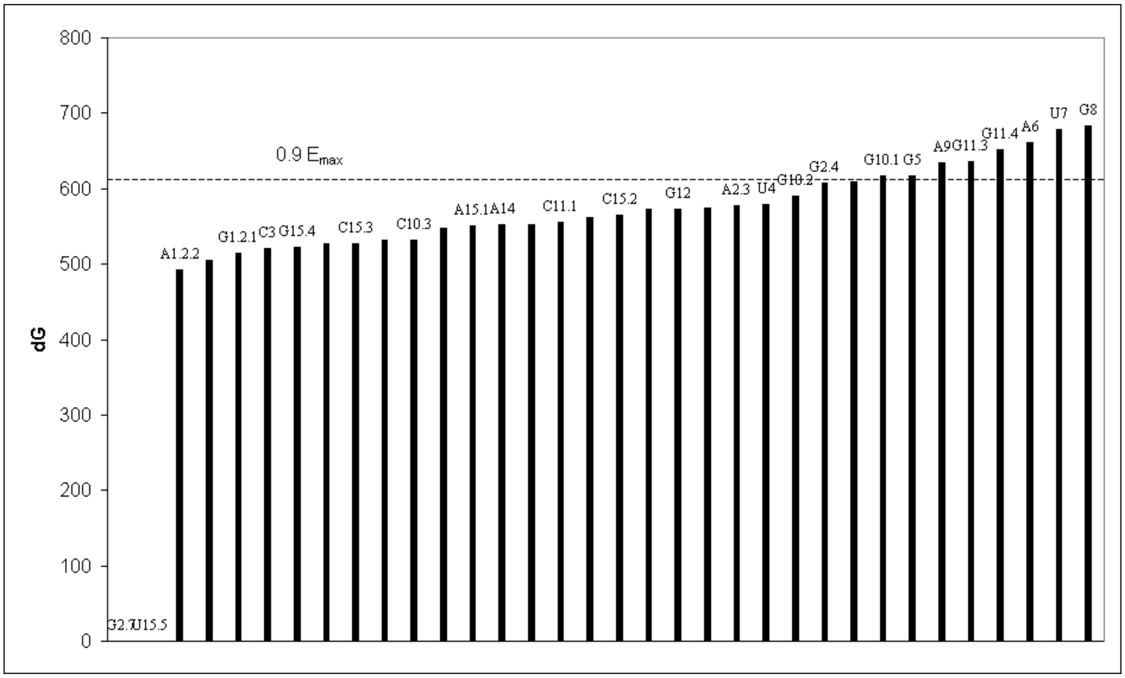

**FIGURE 2b**

**FIGURE 2**. A comparison of the results of the various methods of calculation, on the example of hammerhead ribozyme. Energy histograms are shown, approximate locations of the energy cut-off levels are marked with dotted lines. The right part of a histogram corresponds to higher energy values. (a) Explicit solvent and explicit counterions, cut off $2E_{max}$; (b) implicit solvent and implicit counterions model ("standard" model for this study), 0.9 $E_{max}$ cut off; (c) an example of an energy histogram for a protein: PMI (phosphomannose isomerase, PDB 1pmi), ~450-residues. In PMI, all of the residues with the highest energies ("most destabilizing") were in a very narrow region (on the right). All of the residues marked in bold in the figure are known to be functional (with only one "false positive"-His-54). For RNAs the energy histograms, as seen in the above examples, tend to be more continuous and usually lack the sharply distinct narrow regions (of the highest energies).

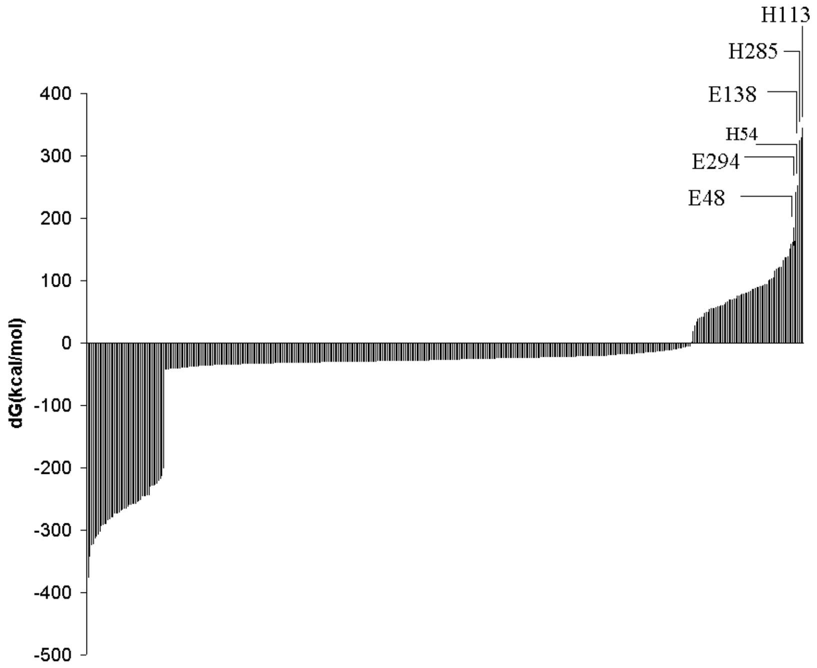

**FIGURE 2c**

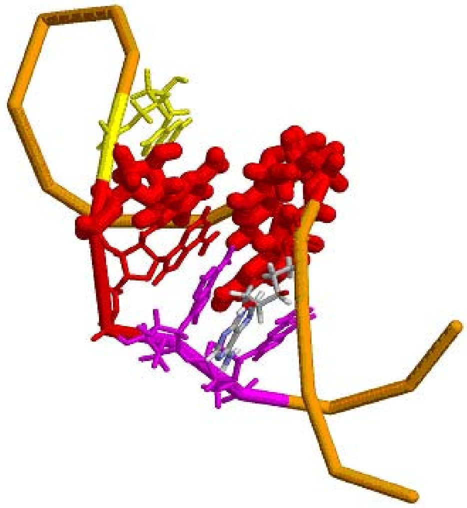

**FIGURE 3a**

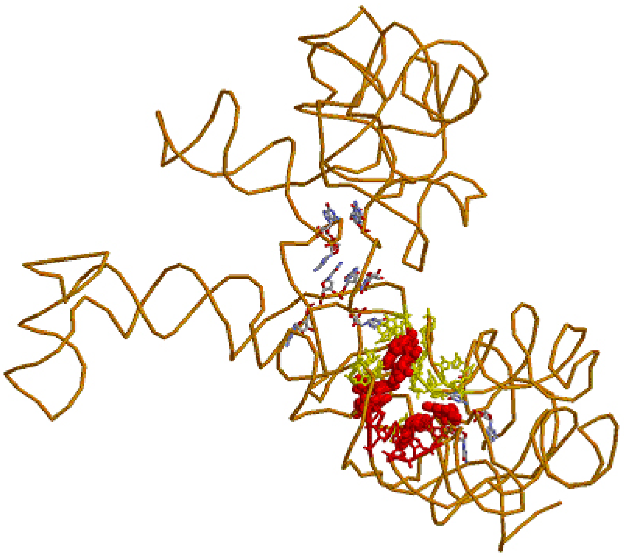

**FIGURE 3b**

**FIGURE 3**. The least stabilizing bases and the known functional residues in ribozymes. The known functional bases are in red (details are in Table 2), the known functional bases that were the least stabilizing are marked using thick lines. Residues less than 4 Å from the active site are in yellow, the rest of the "wire-frame" residues are "false positives". The least stabilizing residues including "false positives" tend to cluster around the active site regions. (a) "Hairpin" ribozyme (PDB 1e4p). A9 stacks with the catalytic G8, residues of the cleavage site (magenta) were also the least stabilizing. (b) 23S catalytic fragment, the nucleotides identified using 0.95 $E_{max}$ cut off. (c) HDV ribozyme (PDB 1cx0), details are in the text. Typically, the "adjacent" nucleotides less than 4 Å from the known functional residues were forming base pairs or stacked with the functional residues. (d) Roles of the least and the most destabilizing nucleotides, active site (red) and protein-binding region (blue) in HDV ribozyme. The protein-binding nucleotides (sequence region 148–153) were the most stabilizing. The least stabilizing residues of the U1A-RBD protein (space filling) that interact with RNA are shown in cyan.

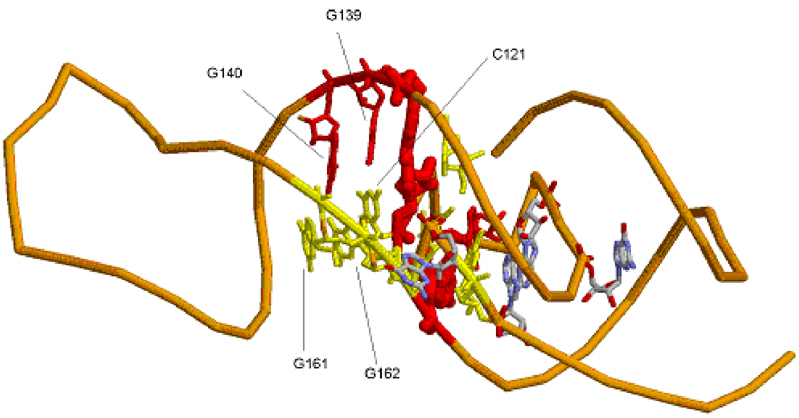

**FIGURE 3c**

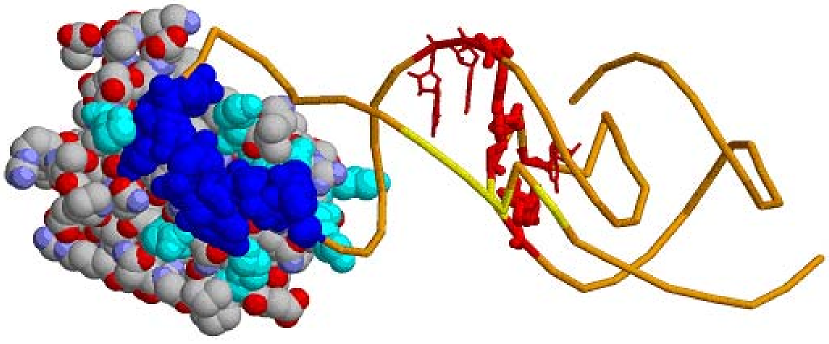

**FIGURE 3d**

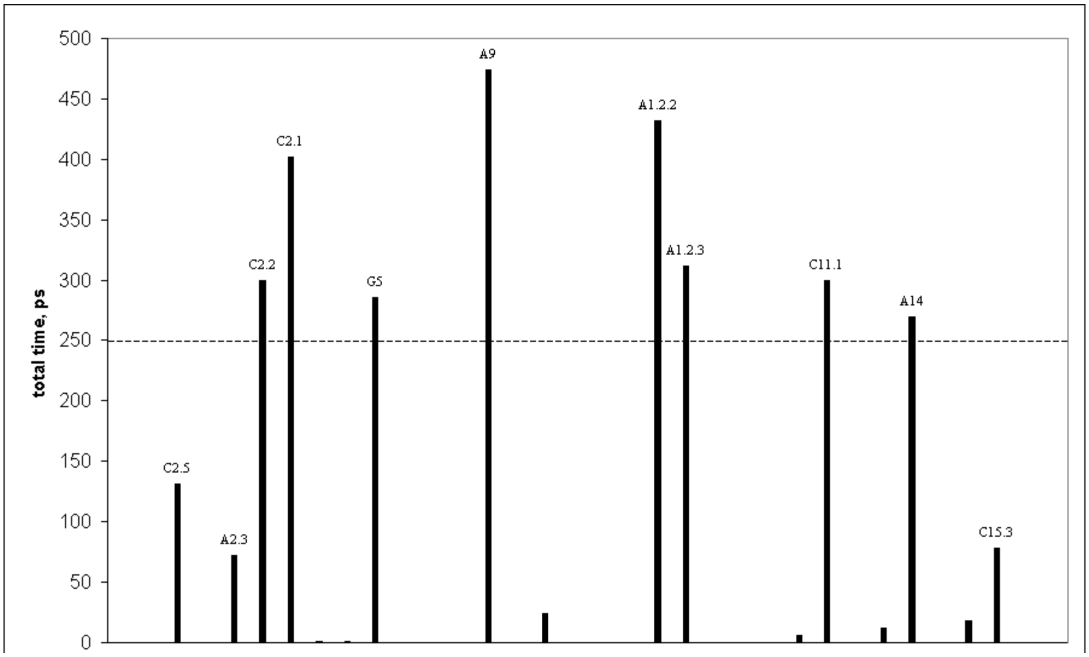

**FIGURE 4**. Results of molecular dynamics of a hammerhead ribozyme represented as an "occupancy profile". The least stabilizing nucleotides were selected using $2E_{max}$ as cut off at each snapshot (1 ps). Total trajectory was 500 ps.

### Modeling Using Explicit Solvent and Counterions and Molecular Mechanics Simulation

The example system for molecular simulations of the hammerhead ribozyme with explicit solvent and counterions included the hammerhead RNA, 34 counterions, and 2,350 water molecules. During molecular dynamics, size of the system was restricted to a spherical box of 35 Å in radius. Each counterion was placed at 3.0 Å distance from each of the phosphates in the backbone. Snapshots were taken each 1 ps. Results of the molecular dynamics simulation are presented using "occupancy profiles" (that can also be drawn by ECMMS routines); for each snapshot the per-base energies were calculated and nucleic bases with energies higher than a cut-off value were marked as "the least stabilizing". The energy cut off for simulations in the system "explicit solvent plus counterions" was 2 $E_{max}$ (corresponds to 0.9 $E_{max}$ in calculations with implicit solvent modeling); $E_{max}$ value was individual for each snapshot. The total time during which a base had energy higher than 2 $E_{max}$ is referred to in this paper as "occupancy" (meaning "occupancy of the region of energies higher than the cut off") and these total time values for each base were used to compile "occupancy profiles" (such as shown in Fig. 4).

### "*In silico*" Mutagenesis

Extensive mutagenesis was performed using MutateMol package (I.Y. Torshin, unpublished). The structure of each mutant was minimized by the same procedure that uses implicit solvent plus counterions model (as above) and then the dG value of the whole RNA molecule was calculated. The value of ddG was defined by subtracting the dG of the native RNA from the dG of the mutant RNA. For exhaustive single-residue mutagenesis, each native base was mutated to the other three types of nucleotides (for example, a native A-base was mutated to G, C, and U). The following two rules were used to distinguish between "mutationally destabilizing" and "mutationally stabilizing" bases: (1) a native base with all of the three ddG<0 (or two ddG<0 and one ddG~0) was labeled as "mutationally destabilizing"; (2) a native base with the three ddG>0 (or two ddG>0 and one ddG~0) was "mutationally stabilizing". Results for td intron mutants are presented in Table 3 and Table 4 summarizes the results of exhaustive single-residue mutagenesis for the 4 small ribozymes.

## RESULTS AND DISCUSSION

When applied to protein structures, calculations of a type considered in this paper show "stabilizing" (dG<0) as well as "destabilizing" residues (dG>0). As show the results of this and previous[14] studies, in nucleic acids all of the bases have dG<0 (using explicit solvent models), thus all of the residues are "stabilizing". However, in the ribozymes studied, the energy of the most stabilizing base was often almost a half of the energy of the least stabilizing base. Such a difference indicates that there are significant differences in the relative energetic properties of the individual nucleic bases in three-dimensional structures of ribozymes.

Taking into account both solvation and configurational components of entropy is important for calculating physicochemically plausible values of the free energy (dG). Therefore, the technique of the calculations first is analyzed and results of calculations using explicit and implicit solvent models are compared. Then the calculation technique was applied to the set of ribozymes (Table 1) and the results are presented in Table 2. Further in this paper, various aspects of calculations such as taking into account configurational component of entropy, comparison of "*in silico*" and "*in vitro*" mutations and application of the method to ribozyme models are considered.

**TABLE 3**
**The Results of the *In Silico* (Computational) and *In Vitro* (Biochemical) Mutagenesis of td Intron**

| Mutant RNA | Calculated ddG | Experimental ddG |
|---|---|---|
| G871C-C946G | 2.8 | 0.7 |
| G871A-C946U | 1.8 | 1.5 |
| C946U | 1.1 | 1.6 |
| G871A | 4.7 | 4.4 |
| A42G -U912C | –1.1 | –0.7 |
| A42G | 0.1 | 1.9 |
| U912C | 0.2 | 1.9 |
| G953A-C966U | 0 | 0. |
| C966U | –0.5 | –0.4 |
| G953A | 0.4 | 0.5 |
| G89A -C854U | 0.1 | 0.3 |
| C854U | 0.9 | 0.1 |
| G89A | 2.0 | 1.9 |

*Note:* ddG=dG(mutant)–dG(native), ddG values scaled to kcal/mol. Correlation coefficient was 0.74; t-test shows the correlation to be significant at 0.995 confidence level. Experimental data are from [44].

**TABLE 4**
**Exhaustive *In Silico* Mutagenesis of Single Nucleotides in Four Small Ribozymes**

| Total Number of Mutations | DdG > 0 | DdG < 0 | DdG ~ 0 | RNA Size | Mutationally Destabilizing Bases | Mutationally Stabilizing Bases | Mutationally Ambiguous Bases |
|---|---|---|---|---|---|---|---|
| | | | | **1e4p Hairpin** | | | |
| 72 | 46 | 19 | 7 | 24 | 3(A7,A9,U16) | 14 | 7 |
| | | | | **1ldz Leadzyme** | | | |
| 90 | 54 | 31 | 5 | 30 | 7(incl.A25) | 9 | 14 |
| | | | | **1hmh Hammerhead** | | | |
| 102 | 98 | 3 | 2 | 34 | 0 | 32 | 2 |
| | | | | **300d Hammerhead** | | | |
| 123 | 116 | 5 | 2 | 41 | 0 | 36 | 5 |
| | | | | **On average for this set of RNAs,%** | | | |
| — | **81%** | **15%** | **4%** | — | **8%** | **72%** | **20%** |

*Note:* Each native base was mutated to the other three, ddG(mutant)=dG(mutant)–dG(native). A native base with all three ddG<0 (or two ddG<0 and one ddG~0) was labeled as “mutationally destabilizing”. A native base with the three ddG>0 (or two ddG>0 and one ddG~0) was “mutationally stabilizing”. The results show that most of the bases are “mutationally stabilizing” (that is, a mutation, in general, is likely to lower RNA stability and only few of the mutations would improve the stability of the native RNA).

### Implicit and Explicit Solvent Models

How do the results of the calculations using implicit models compare with the results obtained through explicit modeling? Because explicit solvent modeling grows computationally expensive with the size of the system, such comparison was made only for the four smallest ribozymes (hairpin, leadzyme, and the two hammerhead ribozymes in Table 1). The results for a hammerhead structure (PDB 1hmh) are analyzed in detail and then the general conclusions on the solvent modeling are presented.

Comparison of the results of energy calculations on the example of hammerhead ribozyme using implicit solvent/counterions and explicit solvent/counterions is presented in Fig. 2. First, all per-residue energy values were greater than zero using implicit solvent while all the values were less than zero using explicit solvent. Second, the known catalytic residues tend to cluster at the regions of higher energies (right sides of the histograms in Fig. 2). The catalytic bases in RNA are not "most destabilizing" (in contrast to proteins with distinctly stabilizing and destabilizing residues [21,22]), but rather "the least stabilizing". Third, energy histograms obtained in systems with explicit solvent plus explicit counterions tend to be less continuous than modeling with implicit solvent (compare Figs. 2a and 2b). Discontinuity of the energy histogram is characteristic for proteins (unpublished data: for example, PMI, Fig. 2c).

Thus, using the explicit solvent/counterions model shows the relative energetic differences between individual bases much more clearly than using the implicit solvent model. Nevertheless, using explicit solvent and explicit counterions produces the results that are not much better (in terms of the number of catalytic nucleotides identified) than those obtained using the implicit modeling. For example, using explicit solvent (Fig. 2a), the nucleotides over the cut off were **G8**, U7, A6, G11.4, A1.2.4, **G5**, U4, **G10.1;** for implicit solvent (Fig. 2b), the nucleotides were **G8**, U7, A6, G11.4, G11.3, **A9**, **G5, G10.1** (known functional bases are in bold font). Quite obviously, there are different energy-cut off rules for different solvent modeling: 0.9 $E_{max}$ for calculations with implicit solvent (Fig. 2b) and $2E_{max}$ for calculations with explicit solvent and counterions (Fig. 2a).

These observations were also valid for the other three small ribozymes (hairpin, leadzyme, and another hammerhead structure). In short, although implicit "solvent damping" does not take into account a number of dependences such as the dependence of "damping" on the distance of the charges from the solute/solvent interface[48], the relative ranking of the per-residue energies obtained using the implicit modeling is close to the results of calculations using the explicit modeling. In other words, results produced by the implicit solvent model and the explicit solvent and counterions are similar in terms of the identified nucleic bases, as it is illustrated by the example of the hammerhead considered above. The role of water in molecular events and enzymatic active sites in a low-water environment is not well understood[49]. However, it is interesting to notice that hairpin ribozyme (and, to some extent, hammerhead ribozyme) catalyzes RNA cleavage in partially hydrated RNA films[49]. Catalysis is minimal but still occurs under conditions of extreme dehydration; mutations of the known catalytic bases of ribozyme abolish catalysis in the dehydrated RNA films. Therefore, calculations using the implicit solvent model not only produce plausible results in terms of the identified bases, but also might model an actual physical process: ribozyme catalysis under conditions of extreme dehydration.

Thus, calculations using implicit solvent or those performed using an explicit solvent model have similar relative ranking of per-base energy values (Figs. 2a and 2b). However, calculations with implicit solvent are considerably simpler and much faster. The results of calculations using the implicit solvent/counterions modeling were used to compile Table 2 for all ribozymes in this study.

### Application of the Calculations with Implicit Solvent to the Set of Ribozymes

The test set (Table 1) includes well-characterized ribozymes of diverse structures and sizes. Many of the functional nucleotides in these ribozymes have been identified by biochemical methods[6,7,8,9, 10,11,12,13]. The results of the energy calculations are summarized in Table 2. In these ribozymes, more than half of the known catalytic residues can be identified by the energy calculations with implicit solvent. On average (Table 2), the 0.9 $E_{max}$ cut off selects less than 20% of all the residues in the entire molecule (depending on the size of the molecule) and most of the known functional residues had energies higher than the cut off. In the structures these "least stabilizing" residues were located in RNA loops as well as in RNA helices (examples are in Fig. 3).

In the 4 small ribozymes (24–34 nt, Table 1), the calculations select up to 8 least stabilizing nucleotides, almost one-third of these RNA structures. Given such a large proportion of nucleotides selected, some of the catalytic ones may be picked up by chance. However, comparison of the sets of nucleic bases selected using the energy calculations with random sets of the same sample size (6–8 nt) shows that the results (Table 2) are statistically significant. Even in the case of the smallest ribozyme (24-base hairpin), 95% of the 1,000 of random 7-nt subsets of the nucleic bases pick no more than 1 of the known functional residues with the false positives randomly distributed over the molecule. The energy calculations, however, correctly identify 5 of the known functional bases in this ribozyme (Table 1). On average for these 4 small ribozymes, the energy calculations are over 10 times more probable to identify a functional base compared to a randomly generated set of nucleic bases. In all of the structures, the least stabilizing residues (including “known”, “adjacent”, and “false positives” in Table 2) typically were placed less than 10 Å from the active sites with very few exceptions (for example, 23S fragment, Fig. 2b), while in the small ribozymes, the “false positives” were less than 6 Å from the active site regions. On average for the set, the majority (~70%) of the least stabilizing residues were either known to be functional or were less than 4 Å from the known functional residues (Table 2). The percentage of “other” or “false positive” was relatively small (5% of all the residues in a molecule on average for the set and no more than 10% for an individual structure). For example, the following nucleotides within catalytic core of a hairpin (PDB 1m5k) were identified biochemically as essential: G8, A9, A10, G21, A22, A23, A24, and C25[31,32]. Half of them were among the “least stabilizing” (Table 2). The “adjacent” nucleotides A40, U42, and A43 (also calculated to be least stabilizing) form base pairs with the catalytically important A23, A22, and G21. Mutations of the A40, U41, U42, and A43 are, actually, known to affect catalysis (at least tenfold drop in activity was observed[31]). Thus, among the 12 residues identified by the methods as “least stabilizing”, 8 nucleotides are known to be important for catalysis and only 4 are “false positives” (no known function).

### Molecular Dynamics Simulation of Hammerhead Ribozyme

During a 500-ps molecular dynamics simulation, only 11 out of the 34 bases in hammerhead ribozyme had energies higher than 2 $E_{max}$ ($E_{max}$ values were determined separately for each shot) at least 10% of the time of the simulation (500 ps, Fig. 4). Out of the 11 bases, 8 bases were “least stabilizing” not less than 50% of the time of the simulation, these 8 bases were catalytic G5 and A9; magnesium-binding A14; bases C2.2 and C2.1 of substrate-binding strand; and also A1.2.2, A1.2.3, and C11.1. Comparison of these results with the results of calculations on the crystal structure (Table 2) shows that catalytic G8 and G10.1 are the least stabilizing only in calculations on the crystal structure and not during molecular dynamics. The bases G5 and A9 are the least stabilizing most of the time during molecular dynamics as well as in calculations on the crystal structure. This observation suggests that the bases G5 and A9 preserve their status of being “the least stabilizing” most of the time during the simulation. Comparison of the bases obtained in calculations on crystal structure and after 500-ps molecular dynamics also allows to eliminate all of the “false positives”; only G5, A9 were identified as the least stabilizing both in the calculations on the crystal structure and during molecular dynamics. Both G5 and A9 are known to be important for catalysis[9,10].

### Correlation Between *In Silico* and *In Vitro* Mutagenesis of td Intron

It would be interesting to compare the results of the method described here with some biochemical data on stability of ribozymes. This was done for group I td intron for which a number of the biochemical studies on stability measurements were done[44,45]. Although no structure of td intron was solved, a biochemically validated model of the catalytic core of the ribozyme is available[37]. Biochemical mutagenesis of a number of bases in this ribozyme was done[44,45] and for some mutant RNAs, dG values were calculated from absorbance-temperature profiles[44]. Comparison of the calculated (on the base of the model[37]) and experimental ddG for each mutant (Table 3, [44]) shows a correlation coefficient of 0.74. According to t-test, the correlation between experimental and calculated ddG values is statistically significant at the confidence level of 0.995. In some cases (mutants G871C-C946G, A42G, U912C, and C854U in Table 3), calculated ddG do not fit well with the experimental data. However, the calculations at least reproduce the right direction of the change in dG and this suggest that such *in silico* mutagenesis can produce physically plausible results. Therefore, this computational procedure was used for extensive *in silico* mutagenesis as follows.

### Exhaustive Mutagenesis of Single Nucleotides *In Silico*

As *in vitro* (biochemical) mutational data on ribozyme stability are scarce, this paper presents results of an attempt to do an exhaustive single-nucleotide mutagenesis *in silico* (that is, computationally). The results of this *in silico* mutagenesis (Table 4) show that most of the mutations (81%, on average) have ddG (dG[mutant] – dG[native]) greater than zero (greater than +0.5 kcal/mol, actually). In other words, a mutation of a native nucleic base in ribozymes to any other base is more likely to destabilize the RNA molecule than to stabilize it. It is interesting to note that in the case of the catalytic core of td intron, analyzed above, experimental and calculated dG for two mutants (A42G-U912C and C966U) were slightly less than zero. However, the melting temperatures of all of the experimentally studied mutants of td intron (those described in [44,45]) were lower than the melting temperature of the native RNA (that is, all of the residues mutated in [44,45] could be labeled as "mutationally stabilizing"). This result (81% of mutations being destabilizing) correlates well with the general notion that most of the mutations of individual bases would disrupt the native packing and thus are bound to be destabilizing.

From the stability of the mutant, the stabilizing properties of the native base usually can be inferred; for example, if mutation destabilized the RNA, then the native base was "mutationally stabilizing". However, mutations of "side-chain deletion" type (like Arg→Ala etc. in proteins) are not possible in nucleic acids. Therefore, each native base is to be mutated to the other three possible bases. To ascertain that the native base was "stabilizing" or "destabilizing" (mutationally), each of the three mutants should be more stable (or less stable) than the native RNA. Otherwise, if one mutant is distinctly less stable than the native RNA, another mutant is more stable, and the third mutant has almost the same stability as the native RNA, mutational studies cannot allow any conclusion as for stabilizing properties of such a native base. For example, in these *in silico* experiments, mutating A21 in 34-base hammerhead (1hmh) to G21 produces a more stable mutant RNA than the native (ddG = –1.5 kcal/mol). However, mutants A21U and A21C are less stable (ddG values are 5.1 and 3.3 kcal/mol, respectively). Therefore, it cannot be concluded that A21 in hammerhead is either "mutationally stabilizing" or "mutationally destabilizing" base. The same ambiguity holds for approximately 20% of the RNA bases (on average for the 4 ribozymes in Table 4).

The exhaustive single-nucleotide mutagenesis also shows that the bases that are unanimously "mutationally destabilizing" (like A7 in 1e4p hairpin — each of the three mutants A7G, A7C, and A7U is more stable than the native RNA) are not very numerous: 8%, on average. In the 2 smallest ribozymes (1e4p hairpin and 1ldz leadzyme), some of the catalytic residues were found to be "mutationally destabilizing" (A7 in hairpin, A25 in leadzyme) in this *in silico* mutagenesis. However, most of the RNA bases (72%, on average for the 4 ribozymes), whether they were catalytic or not, are rather "mutationally stabilizing"; that is, a mutation of such a base in a native ribozyme will definitely decrease the RNA's stability (Table 4). This result suggests that measurements of the *mutant* stabilities cannot always be compared adequately with the calculations of *relative* energetic contribution of each native base to stability of the native RNA. In other words, not only mutation of a catalytic base, but also any mutation of almost any base, proves to be destabilizing for the native structure. In this sense, most of the bases in a ribozyme observably stabilize the RNA (as follows from from mutational experiments) and this observation could lead to a seeming contradiction with the main subject of this study: that functional bases in RNA residues are the least stabilizing. To eliminate this seeming contradiction, it is necessary to distinguish between "*mutationally* destabilizing bases" (which are identified by altering the native structure) and "*relatively* destabilizing bases" (those having the highest energies in the intact, native structure). These "relatively destabilizing bases" are referred to as "the least stabilizing bases" throughout the text of this article. It is the analysis of the *relatively* stability of the bases that identifies half of the known catalytic bases (Table 2 and Figures). It should also be noted that changes in overall ribozyme stability after a mutation (as shown by these "*in silico*" mutations) could not be used to distinguish between catalytic and any other bases (with the few exceptions, like A7 in the small hairpin, Tables 2 and 4). However, in the context of the present study, the term "the least stabilizing" implies "in comparison to the other bases in the native structure" and not "in comparison to mutant RNAs". In other words, in proteins, the *relative* (in calculations on the native structure, such as in [21]) and the *mutational* (in comparison to a mutant) destabilizing properties of individual residues are apparently identical[20,21,22], while in ribozymes, relative and mutational measures for residue contribution to RNA stability distinctly differ. Nevertheless, the method of calculations of *relative* energies described here produces a useful result: identification of many known catalytic and other functional bases using solely structure of a ribozyme molecule.

### Catalytic and Other Types of Functional Residues

The calculations identify as the least stabilizing not only the known functional nucleotides, but also other nucleotides. These other nucleotides are separated in Table 2 into two groups: "adjacent to active site" (less than 4 Å from the known functional residues) and "other" ("false positive"). Although marked as "false positives", in all of the structures these "false positives" were typically placed less than 10 Å from the active sites. Many of the "adjacent" residues were located in the active site regions and were involved in base-pair interactions with the functional residues or at least had hydrogen bonds with them. For example, in 1cx0B (HDV ribozyme), the adjacent residue C121 forms a base pair with the catalytic G139, while G162 forms a hydrogen bond with catalytic G140. The adjacent G161 has hydrophobic (Van der Waals) interaction with the catalytic G140 (Fig. 3c). Another example: metal-binding site in P4 fragment of RnaseP is known to be important for catalysis by RnaseP[7]. The bases G5, G9, C20, and G21 of the metal-binding site were among the least stabilizing, as well as C19 that forms base pair with C7 of the metal-binding site. Only two false positives were identified: A4 and A18.

The least successful method was applied to the catalytic core of the group I intron (P4–P6 fragment, PDB: 1gidA). This structure is not listed in Tables 1 and 2 as it is a fragment of the catalytic core and also because the biochemical data on the functional nucleotides are more complex, so the data are rather discussed below in some detail. About 15% of the nucleotides in the structure were found to be the least stabilizing using the 0.9 $E_{max}$ cut off. Although the known catalytic residues A114 and A207 were not identified among those, a least stabilizing A113 has hydrogen bonds with both catalytic A114 and A207. Another interesting feature was that the other (noncatalytic) functional residues were calculated to be the least stabilizing. For example, among known magnesium-binding residues (A173, A171, G163, U249, G250, A184, G188, G112, G119, U202, and U120[28]), the nucleotides G112, A171, A184, and G188 were the least stabilizing. Among the residues essential for core packing of this ribozyme (A183, A186, C137, G181, G164, and C217[28]), the G181, G164, A186, and A183 were the least stabilizing. The remainder (about 15) of least stabilizing bases were clustered in the central region of the RNA molecule around the known magnesium-binding and core-packing bases. Only 5 bases were far outside the active site region: G129, U253, G254, C255, and U155 (which binds a cobalt hexamine ion). Thus, although the method did not identify the 2 known catalytic residues, the results show that residues important for other functional aspects of the ribozyme were the least stabilizing.

For the P4–P6 fragment, the comparison of the energetic and surface accessibility calculations was made. Although most of the above-mentioned functional residues had significant surface accessibility[28], the surface accessibility calculations identify over 50 residues (~30%) as potentially important. In the case of a large structure (like catalytic fragment of 23S ribosome subunit, for example), more than half of the 498 residues would be “surface accessible”. Therefore, surface accessibility calculations could be useful for finding potential functional residues in smaller structures. Surface accessibility can also be considered as a simplified measure of short-range (2–5 Å) Van der Waals interactions, which neglects long-range (up to 15 Å) electrostatic interactions.

Noncatalytic, but still functionally important, residues were also identified in other ribozyme structures. In 1fg0 (the catalytic fragment of 20S ribosome subunit entirely comprised of RNA[13,29]), at least some of the “false positive” residues (Table 1) are known to be functional though they are far from the catalytic site. C2280 and G2281 interact with K48 of ribosomal protein L15P; G2279 interacts with D49 and S53 of ribosomal protein L44E; U2462 and U2463 interact with R69, G70, and G71 of ribosomal protein L15[30]. These and other residues given in Table 2 were identified using the 0.95 $E_{max}$ cut off. Taking cut offs of 0.9 $E_{max}$ and 0.95 $E_{max}$ for this ribozyme identifies an almost identical set of known functional residues, but the number of “false positives” was reduced significantly using this slightly higher cut off (0.95 $E_{max}$). In 1grzA (group I intron), the active site is not definitely known[12]. However, three of the four proposed potential catalytic residues (A306, A261, A207, C208[12]) were least stabilizing (A207, A261, and A306), G112 forms base pair with potential catalytic C208. A256 and G257 of the domain-domain contact are also important for magnesium binding essential for the catalysis[12]. It also may be of interest to note that the cleavage site nucleotides in the ribozymes analyzed here were not, typically, least stabilizing; only in one RNA (1e4pA, “hairpin” ribozyme), G5 and A6 of the cleavage site were calculated to be least stabilizing (Table 1).

### Other RNA Types and Roles of the Least Stabilizing and the Most Stabilizing Residues

To test the range of applicability of the method, several RNA types other than ribozymes were investigated: AMP-binding RNA, P4 fragment of RnaseP, and protein-binding RNAs (HDV ribozyme, sa50 and 4.5S RNA domain IV). With the exception of the AMP-binding RNA and P4 fragment of RnaseP (already mentioned above), this application was not as successful compared to the results for ribozymes. However, several interesting results are worth mentioning. In 1am0 (AMP-binding RNA), the known AMP-binding residues are G7, G8, A10, G11, A12, G17, U18, and G34[33]. The residues A10, G11, A12, and G34 were the least stabilizing along with “other” A13 and U16. Interestingly, the functional G7 and G8 were the most stabilizing (having the lowest energies among all residues in the molecule). The most stabilizing residues can be functional in proteins ([21] and unpublished data).

The most stabilizing residues also proved to be functional in the two protein-binding RNAs described below. HDV ribozyme (analyzed previously) has a protein-binding region that is 20–30 Å from the catalytic site (Fig. 3d). The binding site of the U1A protein in HDV ribozyme is formed by A148, U149, U150, G151, C152, A153, and G158, and all of these residues except G158 were the most stabilizing (had the lowest energies in comparison to all the other nucleotides). According to the three-dimensional structure[11], these most stabilizing residues of RNA interact with the least stabilizing amino acid residues of U1A spliceosomal protein (K50, R52, K20, K22, K80, R83, and K88). These data obtained from the separate structures of RNA and protein can be used to reconstruct a RNA-protein complex in the absence of a solved structure of such a complex. The SRP (signal recognition particle) assembly has a crucial role in targeting secretory proteins to the rough endoplasmic reticulum membrane. In the structure of 7SL RNA of the sa50 SRP ribonucleoprotein particle, the RNA interacts with proteins at two sites: site-1 (C121, C122, G124, U125, A126, and C129) and site-2 (G111, G113, G114, U135, and A136)[34]. Analysis has shown that C121, C122, and A126 of site-1 as well as G113, G114, and A136 of site-2 were the most stabilizing residues.

The examples of HDV ribozyme and the 7SL SRP RNA show that the nucleotides interacting with proteins can be *not* the least stabilizing *but* the most stabilizing. However, this observation does not appear to be a general rule even in this rather limited set of examples. In 4.5S RNA domain IV (PDB: 1duh)[35], the known protein-binding nucleotides (**A39, A47, G48,** and **G49**, also indicated in bold in the following text) are not energetically distinct from the other nucleotides, while the nucleotides in the immediate vicinity of the known protein-binding residues (C62 forming base pair with **A47,** G61 pairing with **G48**, and A60 pairing the **G49**) were the least stabilizing. In the 23S catalytic fragment, some of the nucleotides interacting with the ribosomal proteins were also among the least stabilizing. Thus, while many of the known catalytic as well as metal-binding and substrate-binding nucleotides in the ribozymes were unequivocally the least stabilizing (Table 2), the protein-binding nucleotides can be both least stabilizing and the most stabilizing.

### Predictions for Model Ribozyme Structures

A model of three-dimensional structure of a ribozyme can be made in the absence of X-ray or NMR data either by using another physical method or a prediction technique. For example, there is a model of hammerhead ribozyme based primarily on fluorescence measurements[36]. The structure itself does not provide any indications where the active site might be located[36]. However, the structural and biochemical data on other hammerhead ribozymes (1hmhA and 300dA in Tables 1 and 2) suggest that the catalytic residues in this hammerhead model would be among **C6 , G8, A9, G11, G13, A28, U43,** and **C44**. Application of the calculation procedure (0.9 $E_{max}$) identifies **G8, A9,** and **U43** as the top-most least stabilizing bases. The other least stabilizing bases were: U7, U10, C24, G25, and A45. U7 has Van der Waals contact with **C6**; U10 corresponds to a magnesium-binding site in a structure (PDB: 300dA). C24 forms base pair to **G13**, G35 is hydrogen bonded to potentially catalytic **G11**, while A45 is the potential cleavage site. No other residues were identified using 0.9 $E_{max}$ cut off.

The method was also applied to models of larger ribozymes. In the 307 nucleotide model of catalytic core of the td group I intron, neomycin is bound in the potential active site[37]. The base C870, known to be catalytically important[37], is located at the neomycin-binding site. Other bases forming Van der Waals contacts with neomycin were: G871, A872, U940, A941, U942, A943, G944, U945, C946, U1057, and G1058. Out of the 307 bases, 18 were identified as the least stabilizing using 0.95 $E_{max}$ energy cut off; 7 out of the 18 nucleotides were forming the neomycin-binding site (catalytic C870, G871, and UAUAG 940-944). The rest of the least stabilizing bases (AAA 46-48, GCCUAA 864-869, G938, and A939) were approximately 10 Å from the neomycin-binding site in the model.

*E. coli* ribonuclease P[38] is a ribozyme responsible for maturation of the 5' ends of tRNAs. The potential catalytic region is formed by A98, A99, A118, A351, and A352[38]. The energy histogram for the 373-base model of this ribozyme was more discontinuous than those shown in Fig. 2 and because of this discontinuity, only 12 out of the 373 bases were identified as the least stabilizing using 0.95 $E_{max}$ cut off. Potential catalytic A118 was at the top of the list of the least stabilizing residues. Most of the least stabilizing nucleotides (excluding G229, G230, and U360* that are likely to be “false positives”) were within 6–10 Å of the potential active site, those were: G68*, G72, G91, G101, G120, G356*, G357*, and U359. Nucleotides invariant among bacteria (according to [38]) are marked with asterisk. Thus, application of the method can predict potential active sites using structural models of ribozymes.

### CONCLUSION

In this study, the relative energetic properties of the nucleic bases were analyzed in experimental structures of ribozymes. Typically, at least a half of the known catalytic residues can be identified using the spatial structure alone without any additional data such as sequence comparison or biochemistry. Results of prediction for structural models of ribozymes are in agreement with the biochemical data on the potential catalytic regions of those ribozymes. In distinction from proteins (functional residues tend to destabilize the proteins[21,22]), the catalytic and other functional bases in ribozymes do not destabilize the RNAs but provide the least stabilization possible (in comparison to the rest of the bases). Two major problems are to be dealt with in further research. First, different types of functional residues were identified in the 8 ribozymes. Those included both catalytic and magnesium-binding residues, thus there is the problem of distinguishing between the different functional types of residues and eliminating the "false positives". A potential solution may be an improvement of the RNA force fields. "False positives" can also be excluded by analysis of the spatial clustering of the least stabilizing nucleotides; a cluster of nucleotides is more likely to correspond to a functional site than an isolated nucleotide. Comparison of the calculations using implicit and explicit solvents can also contribute to removing the "false positives". Second, the flexibility of ribozymes is much higher than that of protein enzymes and large conformational changes are likely to occur during the ribozyme catalysis[28]. In other words, catalysis by RNA may not require a precisely packed structure. Therefore, the kind of calculations presented in this paper can predict functional nucleotides using structural models of ribozymes[36,37,38,50,51], as it was shown on the published models of the three ribozymes.

### ACKNOWLEDGMENTS

I would like to thank Prof. Yu. V. Torshin for helpful discussions, his support, and advice in writing the manuscript.

### APPENDIX I. ECMMS: A LIBRARY AND A PROGRAM

ECMMS ("Energy Calculator for Macro-Molecular Structures") is a library of routines for molecular mechanics calculations. The routines are written in Borland/FreePascal. The main idea behind ECMMS is the possibility to make computationally efficient executables for highly specialized types of molecular mechanics calculations (an example of source code for such an executable is given below). ECMMS routines support graphic output of the energy histograms/profiles (Figs. 2 and 4 of this paper, for example). Composing computationally efficient and specialized executables (that is, stand-alone applications) for a particular research project is distinct from more generous scripting approach as implemented in ICM[52], AMMP[53], and TINKER[54] as well as in many other widely known commercial programs. Another distinctive feature is extreme simplicity of the interface of the executable. No tedious conversion (which is often prone to errors) into any specific format is necessary. For example, the ECMMS-based program used in this study used only two input parameters: a filename of an input PDB file and a filename for the output list of per-residue energies.

ECMMS currently uses UFF+AMBER calculation scheme[24,25,55,56,57]. The reason for combining UFF and AMBER was due largely to extraordinary high accuracy of UFF field in calculations on bond and angle terms. The UFF (Universal Force Field) is capable of reproducing most structural features across the Mendeleev's periodic table with errors less than 0.1 Å in bond distances and 5–10° in angle bend[24]. At the same time, AMBER (Assisted Model Building with Energy Refinement)[25,55] is a force field specifically developed for proteins and nucleic acids. AMBER contains values of partial atomic charges obtained from quantum mechanics calculations. These charge values are more plausible physically than the use of partial charges in UFF scheme[53,56,57].

ECCMS allows us to construct computationally efficient executables. For example, calculation of the energy histogram for hammerhead ribozymes (PDB 1hmh), even using the multiple trajectories technique[26,27,58] (as in the script below) takes only 45 sec on a 3Ghz PC computer (using implicit solvent modeling). The procedural "script" or "macro", designed for the purposes of the present study, is shown below. It also may be noted that specialized procedures analogous to "E_residue" in the following program code (that is, the procedure that calculates the per-residue energies) are rarely available in popular molecular mechanics packages.

```
//ECMMS procedure for the present paper
program RibozymeAnalysis;
uses ecmms;
begin
setnucleic(RNA); //ribozymes;
load_pdb(paramstr(1));
linknucleicchainn(_chn[1], _resls[1].i1,_resls[1].i2);
//initial  modeling
buildhydrogens(_chn[1]);
setactive(1,numberofatoms);
seteterms("bonding bending torsional non-bonding");
cjmin(100);
//multiple trajectories and averaging
setsolvation(ImplicitScheme);
storexyz(0); //stores current coordinates in cash(0)
for j:=1 to 10 do
  begin
    restorexyz(0);
    moldy("time=1ps; T=300K"); //molecular dynamics on cash(0)
    cjmin(100);
```

```
    storexyz(j); // store coordinates in cash(j)
  end;
avrgmol(1,10,11); //averaging models 1-10 to cash(11)
restorexyz(11); //copy from cash(11) to "current" (i.e., cash(0))
cjmin(20);
```

**//Calculating energy per-nucleotide**

```
for i:=_resls[1].i1+1 to _resls[1].i2-1 do
    E_residue(i);

WriteEnergyHistogram(paramstr(2));
end.
```
